# Energy constraints on brain network formation

**DOI:** 10.1038/s41598-021-91250-y

**Published:** 2021-06-03

**Authors:** Kosuke Takagi

**Affiliations:** Unaffiliated, Saitama, Japan

**Keywords:** Learning algorithms, Network models

## Abstract

Energy constraints are a fundamental limitation of the brain, which is physically embedded in a restricted space. The collective dynamics of neurons through connections enable the brain to achieve rich functionality, but building connections and maintaining activity come at a high cost. The effects of reducing these costs can be found in the characteristic structures of the brain network. Nevertheless, the mechanism by which energy constraints affect the organization and formation of the neuronal network in the brain is unclear. Here, it is shown that a simple model based on cost minimization can reproduce structures characteristic of the brain network. With reference to the behavior of neurons in real brains, the cost function was introduced in an activity-dependent form correlating the activity cost and the wiring cost as a simple ratio. Cost reduction of this ratio resulted in strengthening connections, especially at highly activated nodes, and induced the formation of large clusters. Regarding these network features, statistical similarity was confirmed by comparison to connectome datasets from various real brains. The findings indicate that these networks share an efficient structure maintained with low costs, both for activity and for wiring. These results imply the crucial role of energy constraints in regulating the network activity and structure of the brain.

## Introduction

Brain functions such as perception and cognition arise from collective activity of neurons supported by a large-scale network^1–5^. The neurons in the brain comprise a network with interconnecting “wires”, in which signals are processed and integrated^6–8^. Regarding brain network formation, the biological processes of neuronal cells and their mechanisms are well studied^9–12^. However, it remains unclear how network formation is regulated to generate characteristic network structures by organizing a large population of neurons^13–15^.

Because the brain is physically restricted and its energy resources are limited, the energy constraints of the brain might be an important factor affecting network formation^8,14,15^. The relationships between energy and brain characteristics (such as size, organization and network geometry) can be found through various experiments and observations^16–23^. They indicate a basic effect of energy constraints on the development and evolution of the brain across different species. This effect arises from changes on a cellular scale, in which the previously described relationships regulate the dynamics and structures of neuronal cells^24–31^. In addition to various biological studies^32–34^, direct evidence that the brain network is optimized with respect to these energy resources can be found in the structure of the brain connectome. For example, they contain large-scale clusters interconnected by hub regions, creating a small-world network structure^7,8,15^, and the activity organized near criticality facilitates signal transfer in the brain^2,35–37^. These clusters improve the efficiency of communication between neurons and brain regions and information processing on the scale of the whole brain. Thus, it can be assumed that the requirement for energy optimization strongly affects network formation in the brain.

Regarding wiring structures such as small-world and scale-free structures, existing models such as the Watts–Strogatz model and the preferential attachment model effectively describe the formation processes causing these characteristic structures^38,39^, yet, in application to the brain network, the mechanisms and the driving force inducing such behaviors are not clear. In contrast, the relation between network structure and energy consumption, for example, would be supported by substantial evidence obtained from biological studies^32–34^. In order to build a generative model incorporating these biological facts, the energy function, the target objective function to be minimized, would be specified^40,41^. Regarding the network energy cost, two types of definitions can be used: the wiring cost, which is the energy needed to construct connections, and the activity cost, which is the energy consumption associated with the signal transfer activity^14,15,34^. On the other hand, plasticity in neural circuits, which alters the connection strength in response to the activity level, is relevant to maintaining brain function by enabling memory formation and learning in an activity-dependent manner^9,10^. Therefore, I introduced a network model with an energy function in a form that correlates the wiring cost and the activity cost. The model was implemented based on this function using artificial neural network methods^42,43^.

## Results

### Network model

The network can be described by a connectivity matrix $W=(w_{i,j})$, where $w_{i,j}$ is the connection strength between nodes *i* and *j*. For connectome datasets from the real brain, nodes correspond to brain regions or neurons, and the connectivity is measured by means of various devices. The examples compared in this paper are listed in Table 1 which contains a wide variety of datasets across different species.

**Table 1:** Definitions of the connectome datasets.

Connectome type	Node type	Number of nodes	Connection type
Human functional^44^	Regions	177	Functional connectivity
Human anatomical^44^	Rregions	188	Axonal fibers
Cat^45^	Regions	65	Axonal fibers
Worm^46^	Neurons	272	Synaptic connections
Fly^47^	Neurons	1781	Synaptic connections

A description of each connectome is listed in this table. Regarding the network nodes, the type (neurons or brain regions) and the total number are given. Additionally, the table contains the type of connection used to define the connectivity strength between nodes.

Regarding the energy cost of the brain network, two types of definitions, the activity cost and the wiring cost, can be used^14,15,34^. The wiring cost is the energy expended to create the connections between nodes; in the model, it was estimated by a simple definition, the total connection strength for each node *i*,

1 $$\begin{aligned} E_{w} (i) = \sum _{j} |w_{i,j}|. \end{aligned}$$

The activity cost was taken to reflect the state of each node (activity or inactivity), which varies according to the network activity. The activity associated with signal processing in the brain can be modeled by successive patterns of activation and deactivation through interactions between network nodes, neurons or brain regions. They are stimulated by the external environment and maintained with spontaneous activities^1,3,37^. For the network model with an $N \times N$ matrix, the activation state is represented by the *N*-dimensional vector $V^{0}=(v^{0}_{i})$, and one description of signal transfer in the network is given by $v_{i} = \sum _{j} w (i,j) v^{0}_{j}$, the transition from $V^{0}$ to $V=(v_{i})$^40–43^. Then, reflecting the activation states, the activity cost was defined as

2 $$\begin{aligned} E_{a} (i) = \sum _{j} |v_{i} w_{i,j} v_{j}|. \end{aligned}$$

The activity cost in this form is similar to the Hopfield energy function and those of various neuronal network models, a relevant definition widely applied to brain models and learning models of artificial neural networks^42,43^. In this model, it can be interpreted as the information transfer cost associated with brain activity. In this functional form of Eq. (2), connections between strongly coactivated neurons are dominant, and those with inactive nodes in near zero-activity states are negligible. Thus, excluding the contribution of random noise, it can approximately estimate the energy cost related to information transfer through effective connections within the network.

In the simulation of state transitions, the connections *w* and the states *v* were assigned both positive and negative values, reflecting the excitatory and inhibitory correlations in the brain network^3^. On the other hand, the energy terms were evaluated as the absolute values |*w*| and |*v*| because the net energy consumption is positive. However, when energy consumption is estimated, neuronal activity is assigned a positive value. Positive and negative signs were introduced to distinguish excitation and inhibition in calculating the signal transfer. Accordingly, they totally determine the successive state as $v_{i} = \sum _{j} w (i,j) v^{0}_{j}$, which increases or decreases the activity level in the received node.

Using these definitions, a normalized energy function for each node was introduced as

3 $$\begin{aligned} E_{a} (i)/E_{w}(i), \end{aligned}$$

where the activity cost divided by the wiring cost represents the energy consumption ratio^41^. Based on the assumption that this function is minimized, the network changes the connection weights to minimize the average ${<} E_{a} (i)/E_{w}(i){>}$. By this form of the energy function, this process works to decrease the activity cost or increase the wiring cost by refining the connections. When higher- and lower-activity nodes are compared, the process strengthens the weight connections specifically for more active nodes and weakens them for less active ones. Thus, this definition gives one simple expression for the activity-dependent refinement process mimicking the biological processes of neurons.

A direct observation of neurons in regard to the cost reduction mechanism of the synaptic activity associated with the transmission of information revealed that excessive metabolic cost is avoided^24–26^. Additionally, network patterns are arranged to minimize the wiring cost by adjusting the path lengths and connections of dendrites^16,27–31^. Moreover, cost reduction is completely achieved by optimizing energy efficiency, described as the information transmission per energy cost used, instead of the transmission itself^24–29^. Thus, the biological evidence supports the minimization of the term Eq. (3), which is represented in normalized form as energy efficiency.

### Constraints and stability

In the simulation of the network model, the connection strength *w* was adjusted repeatedly to minimize the normalized energy defined above. The activation state *v*, the other parameter used in the definition for Eq. (2), was taken randomly under the assumption of homogeneity. The external environment, a main source of the input stimulus, would be independent of the internal state of the network.

Because of the physical constraints on the brain, the variables in this model were taken to be restricted in a finite range^40^. Without limit conditions for the variables, the normalized energy form causes instability, divergence or convergence. Under the requirement to reduce energy, two exceptional cases are allowed where the wiring cost diverges to $\infty$, $E_{w} \rightarrow \infty$, and the activity converges to 0, $E_{a} \rightarrow 0$. These are unphysical states, featuring infinitely strong connections or zero-state nodes without reactions. To avoid these cases, the following limit conditions were introduced.

Regarding the wiring cost, an upper bound was imposed on connection strength. Given the boundary $w_{u}$, when the connection strength exceeds $w_{u}$ during the adjustment process, the elements $|w (i,j)| > w_{u}$ are reduced to this value: $|w (i,j)| \rightarrow w_{u}$. On the other hand, regarding the activity cost, the lower limit was taken to prohibit the zero state, where the nodes are identical and inactive. In the refinement process, when the averaged energy $E_{a}$ reaches the lower limit denoted as $E_{a}^{L}$, each element of the connectivity matrix is shifted as $w \rightarrow (E_{a}^{L}/E_{a}) w$. Such an adjustment mechanism, scaling the connection strength in response to activity, is found in the real brain network in the form of homeostatic plasticity^11,12^. An alternative possible mechanism prohibiting zero states would be given by varying the input signal *v* according to the values of the energy; for example, enhancing the signal for small $E_{a} (i)$ nodes. However, based on the assumption of homogeneity of the external environment as mentioned above, a simple model with random input signals was taken in this simulation.

**Figure 1:**
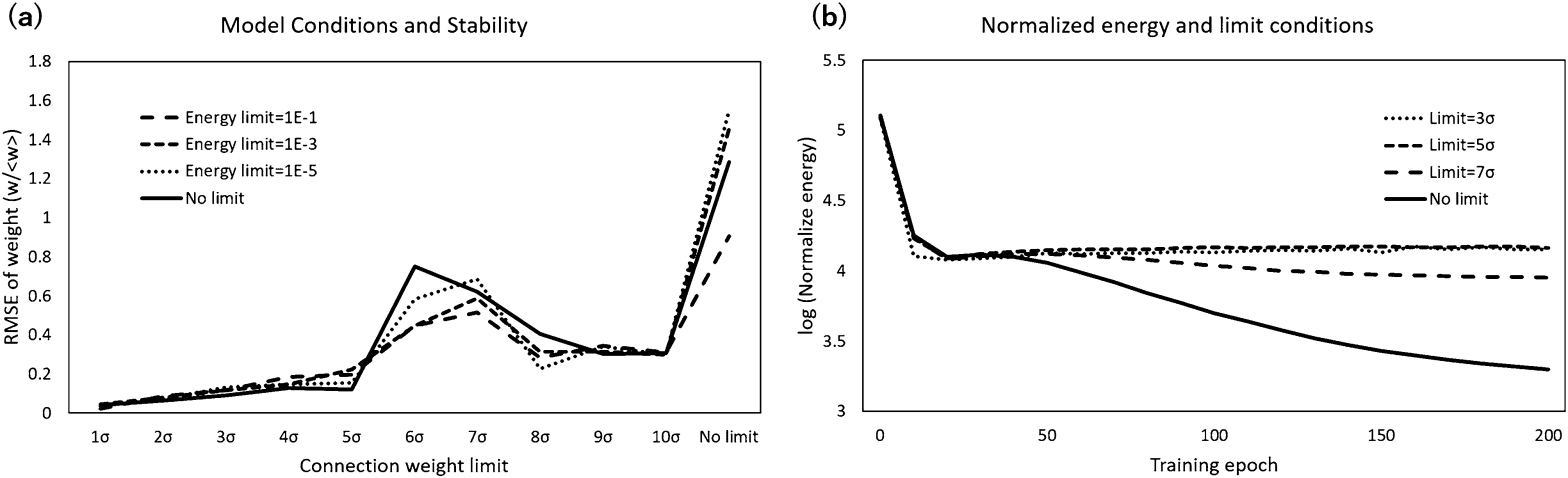
Network stability and energy convergence of the model. (**a**) Network stability. The stability of the network was evaluated by the difference between the networks for each condition. The differences were calculated from the root-mean-square error (RMSE) of the connection weight *w* as explained in the Methods. The upper limit conditions for the connection weight are listed in the horizontal axis and include values form 1 to 10 times the standard deviation $\sigma$ as well as the no-limit case; the conditions for the activity cost were set to $\alpha = 10^{-1}, 10^{-3},$ and $10^{-5}$, where $\alpha$ is the ratio to the initial value as explained in the Results section. The averages across 100 networks for these three conditions are shown as solid, dotted and dashed lines, respectively, on the graph. (**b**) Energy convergence. The estimated values of the normalized energy, the target function for the minimization during the network refinement, are plotted for 200 epochs. The simulation was repeated 100 times for each condition, and the averaged values were taken for each 10-epoch bin. In this graph, the different conditions for the upper bound of weight, $3 \sigma , 5 \sigma , 7 \sigma$ and the no-limit case, were compared at a fixed value of the lower bound for the activity cost, $\alpha =10^{-3}$.

Figure 1a shows that the limit for the connection weight contributes to stability, reducing the difference between generated networks. In the simulation, the upper limit value $w_{u}$ was taken as ${<}w{>} + n \cdot \sigma$ with the average ${<}w{>}$ and the standard deviation $\sigma$ for the absolute values |*w*|. Then, $w_{u}$ was calculated for $n \cdot \sigma$ values of $1 \sigma$ to $10 \sigma$, $n=1, \ldots , 10$ (that is, $n=1, \ldots , 10$) compared to the no-limit case. Additionally, $E_{a}^{L}$ was parameterized as $E_{a}^{L} = \alpha E_{a}^{0}$ with the initial value of the activity cost $E_{a}^0$ and the cases with $\alpha = 10^{-3}, 10^{-5},$ and $10^{-7}$ are taken. Then, the differences were estimated by the root-mean-square error (RMSE) as explained in the Methods. Compared to the case without a limit at the right end of the graph, the cases of the finite limits, $1 \sigma \sim 10 \sigma$, are stable, with small variations of the generated networks under the same conditions. Stable states were achieved at approximately $8 \sigma$; additionally, even in larger limit values such as 8, 9, or $10 \sigma$, the limit condition contributes to the stabilization of the network by prohibiting the exceptional cases caused by the divergence of the wiring strength. While the limit for the activity cost, $E^{a}_{L}$, has no prominent effect on these tendencies.

In the time development of this model, the estimated energy curves confirm that these conditions are effective in preventing the exceptions of divergence and convergence (Fig. 1b). For the fixed limit value of $E^{a}_{L}$ with $\alpha =10^{-3}$, the results for the $w_{u}$ limits with $3 \sigma , 5 \sigma , 7 \sigma$ are shown. Except for the case without the energy limits (the no-limit case), the state stabilized with regard to energy. In the no-limit case, because of the exceptional cases of $E_{w} \rightarrow \infty$, the normalized terms that were given as $E_{a}/E_{w}$ decreased without limit, and no stable state was apparent. Thus, energy constraints, especially an upper limit on connection strength, contribute to stabilizing the networks for each condition.

### Node strength distribution

The distribution shape of the node strength characterizes a statistical feature of this network. Various studies show that brain connectome data exhibit small-world-like network features, with highly connected hubs^2,8,15^. In our model, as a consequence of the refinement process, the node strengths are adjusted according to their activity levels. Especially highly activated nodes with large $E_{a} (i)$ values tend to increase the connection strength $E_{w} (i)$ by dispersing the energy concentrated on these nodes.

**Figure 2:**
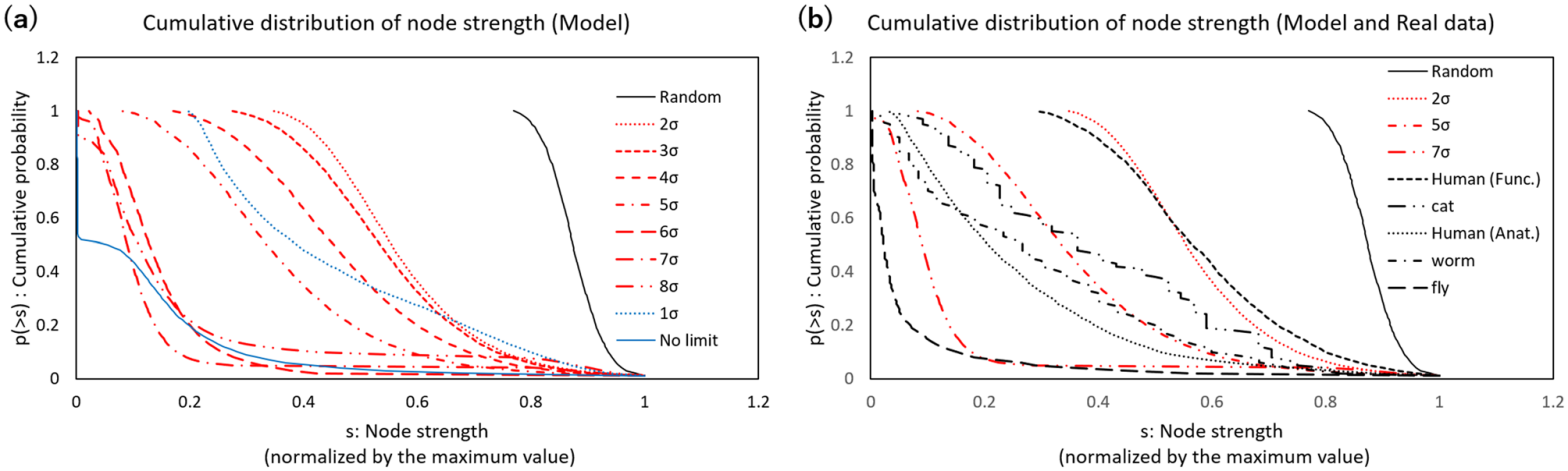
Distribution of node strength. (**a**) Model. The cumulative distribution of node strength, the sum of the connected weights for each node, was plotted. These values were normalized by the maximum value for each network, and then the maximum value on the horizontal axis was fixed at 1. For the model and the random graph, the cumulative distribution was estimated from the results of 100 iterations of the simulation, and each distribution plots 100 points. The upper limit condition for the weight was set at different values form $1 \sigma$ to $8 \sigma$ as well as the no-limit case; for the activity cost limit, $\alpha = 10^{-3}$ was used. The distributions of the model are shown by red lines except for $1 \sigma$ and the no-limit case, which are shown by blue lines. The result of the random graph is shown by the black solid line. (**b**) Model and connectomes. The connectomes of humans^44^ and various other animals (the cat^45^, the worm^46^, and the fly^47^) are overlaid as black dotted and dashed lines, where the distribution of the fly is based on 100 plotted points. The model conditions for $2 \sigma ,5 \sigma$, and $7 \sigma$ were taken from panel (**a**).

The result (Fig. 2a)indicates that the model networks shown in the red lines contain a large number of strongly connected hub nodes, which might be strengthened in the above mechanism, compared to the random network shown by the solid black line. In the random graph, node strength values are distributed in the narrow range as an effect of averaging the connections for each node. While the value range of this model is wider, the difference between the maximum and the minimum value for each condition is large. It is significant, according to increasing of the upper limit, that the distribution shifts to the left on this graph with expanding the distribution range. Additionally, they are compared to the extreme cases shown by the blue lines, where the strict limit of $1 \sigma$ and the no-limit case behave differently, indicating the restriction of the model.

This result shows the long-tailed shape of the distribution, which indicates a relatively large population of strong nodes. The similarity between the real brain networks and these model results is shown in Fig. 2b, where human and animal connectome datasets are included in the comparison. These strong nodes act as hubs, giving the network a small-world structure, which is expected to improve the efficiency of the information transfer by decreasing the distances between the nodes^8,14,15^.

**Figure 3:**
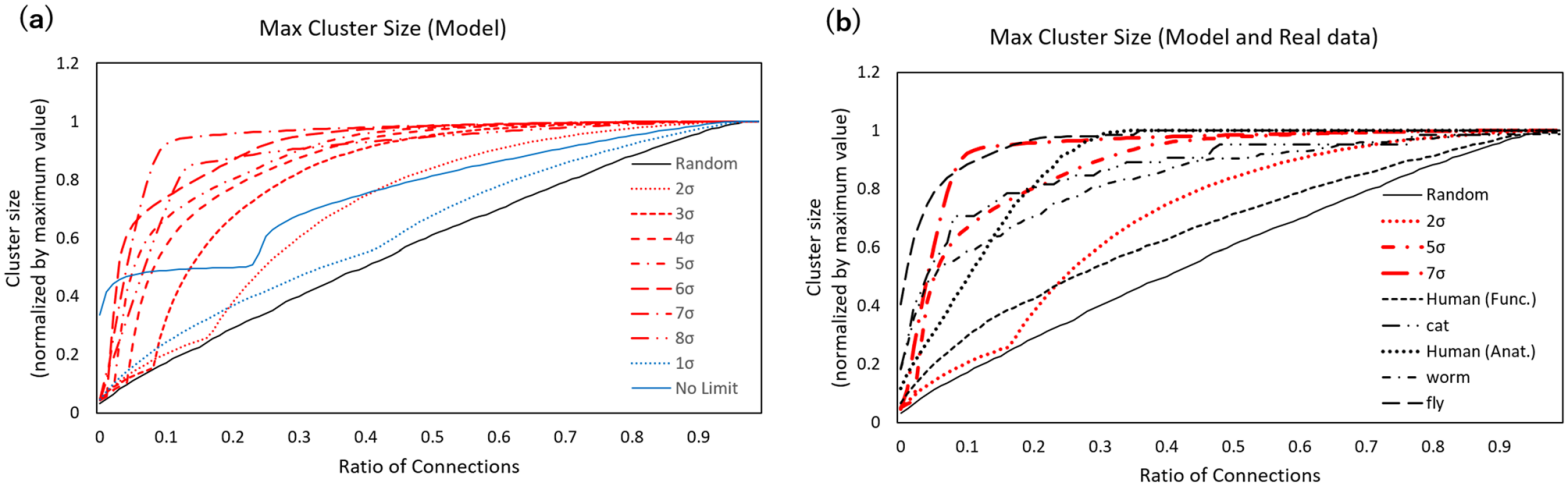
Network cluster size. (**a**) Model. The maximum cluster size of the network was estimated from the same data sets and is shown in the same conditions and style as in Fig. 2a. Subnetworks with strong connections were selected, and the cluster size was estimated for each network. The estimated values were normalized by the value of the initial network. The horizontal axis indicates the ratio of the connection numbers at intervals of 0.01 with which the strong connections were selected according to their values. (**b**) Model and connectomes. The connectomes were compared in the same conditions and style as in Fig. 2b.

### Cluster size

The functional relevance of this model can be shown by the cluster size of the network. Hub nodes produced by the above mechanism induce large population groups (clusters) whose components are connected, and signals are transferred rapidly between components through these hubs. The maximum cluster size in the subnetworks constructed by a small number of connections gives an indicator of the efficiency of cluster formation. I evaluated this indicator while varying the total number of connections as explained in the Methods. The maximum cluster sizes (Fig. 3a) show that, compared to the random graph result, the model network exhibits significantly increased values. Additionally, the connectome datasets shown in Fig. 3b share the same feature, with large cluster sizes. Thus, in addition to the large number of hubs, it identifies another common feature between the model and the connectome datasets across different species.

Because the large cluster size enables efficient communication with small distances between brain regions, it reduces the activity cost associated with signal transfer^2,7,8,15,35–37^. In addition, the same graph (Fig. 3b) shows that these networks, except for the random one, retain large values even with the small connection counts shown on the left side. This indicates that the large cluster sizes of these networks are preserved with a small number of connections. In these networks, the small-world structure can be maintained with a small wiring cost. Thus, the network formation mechanism leads to cost-effectiveness in terms of these two aspects-the activity cost and the wiring cost.

## Discussion

These results show that, based on a simple mechanism, a network model can reproduce multiple features of real brain networks. In this model, the network connections are refined according to the activation level of each node. The active nodes work to strengthen their connections until the network reaches the stable state. This process allows the abundant appearance of strongly connected nodes known as hub nodes, which are commonly observed in various connectome networks^8,14,15^ (Fig. 2). Furthermore, the same mechanism induces the formation of large clusters between coactivated nodes with strong connections (Fig. 3).

This process of cluster formation is cost-effective and of functional relevance in the brain network^2,7,8,15,35–37^. A comparison to the random graph in Fig. 3b shows that the model networks and the connectome datasets preserve large clusters with a small number of connections. Thus, cluster formation is accomplished with low wiring costs. Additionally, because the large size of each cluster enables its components to find short paths to reach others, this feature might help improve signal transfer by allowing extensive communication between multiple regions of the brain at a small activity cost.

The results imply that these network features arise from a common mechanism. In comparison to real brain networks, the network variations for different limit values cover the results of a wide range of connectomes across different species (Figs. 2 and 3). Thus, a mechanism based on a simple principle of energy allocation provides a plausible model reproducing fundamental aspects of the brain network. In addition, the connectomes contain data on different scales from the neuron level to the large-scale cortical level. The present finding has potential applications for a wide variety of brain network studies on topics from neuronal behavior to cognitive processes.

However, to clarify differences between species more accurately, a further extension of the model is required. For example, it is known that the connection numbers for each single node are restricted in the real brain network^27–29^. In contrast to the fully connected network of the simulation model, real brain networks are sparse and have fewer connections per single neuron. By calculating the average of the large ensemble, the variation between single nodes decreases, which contributes to a narrowing of the range of the node distribution, as shown in Fig. 2. This may be negligible for large networks, such as “Fly” in Fig. 2b, and may cause discrepancies with respect to the simulation data. Thus, the implementation of constraints, such as the connection numbers of a single node, is required for future extensions.

Finally, the complexity of the architecture is another important issue to address in the future. Although the current model has a simple single layer, true networks in the brain consist of multiple layers with hierarchical structures. Subsequently, their dynamics are based on synergetic interactions integrating information on external inputs from multiple sources. Additionally, another factor to be considered for the extension of the model would be the heterogeneity that stems from a variety of conditions. For example, the input signal of the simulation, which was taken randomly, should be affected by changes in external environments and diverse sensory responses to these stimuli, which might reflect the features of each local brain region. Furthermore, the coupling between activity and metabolism, which was implicitly assumed to be constant in Eq. (2), could vary according to regional differences such those induced by physiological conditions or brain disorders^21–23^. These factors would be required for further analysis to depict the functional details of each brain region and their impacts on the signal integration processes at the whole-brain scale. Thus these effects may have essential importance, especially in elucidating higher-level cognitive functions of the human brain^4,5^.

## Methods

### Connectome data

The model network was compared to the connectomes of the real brain networks of humans and animals^44–47^. The connectivity matrices in the preprocessed form are publicly available via the web services ‘USC Multimodal Connectivity Database’^48^ (http://umcd.humanconnectomeproject.org/ 14 October 2020, date last accessed) for the human connectome and ‘neurodata’ (https://neurodata.io/project/connectomes/ 14 October 2020, date last accessed) for other animals. For the human functional connectome, the dataset tagged “1000$\_$Functional$\_$Connectomes” was taken from the above site, and data tagged “NKI$\_$Rockland” were used for the human anatomical connectome. As the reference data set, 100 randomly selected files were taken for each connectome dataset. In contrast, the animal connectome information for each species was summarized in a single file. For each dataset, the definition of the node and the connectivity are different, and each network contains a different number of nodes, as summarized in the following table.

### Model implementation

The refinement process was started from a random matrix, the elements of which correspond to the positive or the negative connectivity and take a randomly assigned value drawn from a normal distribution with a standard deviation of 0.5 and an average of 0. In this simulation, the matrix size was set to $N=200$. Additionally, the input signal vector $V^{0}=(v^{0}_{j})$ was selected randomly under the assumption of homogeneity as mentioned in the Results section and was taken as follows. For each signal set, a probability *p* was randomly selected in the range $0< p < 0.5$. Each $V^{0}_{i}$ element equals 1 or $-\,1$ with a probability of *p* and is set to 0 otherwise^41^. For each simulation condition, $10^{4}$ sets of input vectors were used.

The algorithm of this model, explained in the model description, was implemented using TensorFlow with the Adam optimizer^49^. The simulation was repeated for 200 epochs, each of which contained 10 batches, with the learning rate of 0.01. For each repeated batch process, the normalized energy, Eq. (3), of each node was calculated for the weight matrix *W* and a batch set of $10^{4}/10$ input signals, which was randomly selected from the total $10^{4}$ set of $V^{0}$. Taking the average of this energy, the gradient was calculated with respect to the weight elements $w_{i,j}$, the trainable variables of this network model. Then, according to these values, the optimizer adjusted each weight strength $w_{i,j}$ to minimize the energy.

Because an asymmetric matrix *W* was used, the refinement process was also applied to the transposed matrix $W^{T}$ equivalently for each process. Additionally, in order to stabilize the computation, before the refinement process of each epoch, the matrix connections were normalized as $W \rightarrow W/{<}W{>}$, where ${<}W{>}$ is the average of the connection. They were restored by the inverse process, $W \rightarrow {<}W{>} \cdot W$ with the same constant ${<}W{>}$, after the refinement.

After the adjustment process for each epoch, the limit constraints were adapted to the weight matrix. As explained in the Results section, the connection strengths exceeding an upper bound $w_{u}$ were reduced to this value, where $w_{u}$ was calculated with the standard deviation of *w* for each epoch. Additionally, when the activity energy $E_{a}$ reaches the lower limit, the matrix weight is shifted to larger values to avoid the zero state with respect to the activity cost, $E_{a} \rightarrow 0$. These processes were repeated for 200 epochs until the target function decreased sufficiently. The source code of this model is publicly available on GitHub (https://github.com/coutakagi/2020v1/).

### Network stability

In Fig. 1, for the set of networks obtained with the same conditions, the stability was estimated using RMSE between networks. First, weights for each network were ordered according to their strength, $w(0) \ge w (1) \ge \dots$ and normalized by the average ${<}w{>}$ as $w \rightarrow w/{<}w{>}$. Then, RMSE between networks was calculated using these values for each network in the same order.

### Distribution of node strength

The distribution in Fig. 2 represents node strength. It is defined as the sum of the connected weights $$s_{i} = \sum _{j} |w_{i,j}$$| for each node, where the absolute values were taken to be the same as in the case of the wiring cost defined in the model description. All of these values for each condition were ordered: $$s (0) \ge s(1) \dots $$. Then, 100 points were taken at equal intervals, where they are taken at the order nearest to $$ ((n+1)/(100+1)) N$$ for $$n=0 \dots 99$$ with *N* being the total size of the dataset. Then, these points were plotted against the cumulative probability, $$(n+1)/(100+1)$$ for each *n*. For the real brain data shown in Fig. 2b, the same method was used, and 100 points were extracted in each plot, except for the “Cat” data with 65 nodes (Table 1), in which all of the points were plotted.

### Cluster size

The cluster sizes shown in Fig. 3 were determined as follows. For each ratio *r*, the ratio of the numbers of connections, the subnetwork was constructed with $r \cdot n_{c}$ connections, where $n_{c}$ is the number of nonzero connection weights. The connections of the subnetwork were selected according to strength. Then, the hub cluster size was evaluated by the number of nodes connected to a single node. The maximum size of each subnetwork was plotted in Fig. 3.

For the animal connectome datasets, which are given as topological network data, the following methods were used. Because the topological network data give binary values for the connection strength (1 or 0), the connection strength *w*(*i*, *j*) was estimated using the degree as $w (i,j) \propto d(i) d (j)$, where the degree *d* of a network node is the total number of connections. The connections were selected according to these values.
